# Evaluation of novel surfactants for plant transformation

**DOI:** 10.1186/s13104-022-06251-5

**Published:** 2022-12-08

**Authors:** Jennie Huynh, Sara K. Hotton, Ron Chan, Yasra Syed, James Thomson

**Affiliations:** Crop Improvement and Genetics, Western Regional Research Center, USDA-ARS, Albany, CA USA

**Keywords:** Transformation, Surfactant, Arabidopsis, Agrobacterium, Silwet^®^ L-77, BREAK-THRU^®^-OE446, -S200, -S233, -S240, -S279, -S301 and -SP133

## Abstract

**Objective:**

Assess the efficiency of seven novel surfactants, relative to the typical Silwet^®^ L-77, for floral dip transformation of Arabidopsis.

**Results:**

Floral dip transformation of Arabidopsis has been used consistently for 20 years with little change in the protocol. Here we directly compare seven novel surfactants (BREAK-THRU^®^-OE446, S200, S233, S240, S279, S301 and SP133) to the standard Silwet^®^ L-77 for efficiency of Arabidopsis transformation providing an example of how the surfactants can help other plant transformation protocols. Relative transformation efficiencies ranged from − 44 to + 45% compared to Silwet^®^ L-77. Surfactants S200, S240, and S279 demonstrated the greatest enhancement in transformation.

**Supplementary Information:**

The online version contains supplementary material available at 10.1186/s13104-022-06251-5.

## Introduction

Plant biotechnology offers tremendous promise for improving crops by increasing the sustainability and reducing the environmental impact of production. These technologies have led to crop plants with enhanced nutritional properties, abiotic stress tolerance, disease and pest resistance, and other agronomic traits, as well as crop tools for accelerated precision breeding. Although genetic engineering and genomic editing approaches have been applied successfully to various plant species, relatively few crops have an easy and efficient means of transformation, which restricts progress in research as well as agricultural deployment. Thus, additional techniques for broadly and efficiently applying transformation technologies to a wide range of crops and their constituent elite varieties are required.

One of the earliest enhancements to transformation was the use of surfactants, which modify the surface properties of liquids to enhance their spreading, wetting, emulsifying, dispersing, or other characteristics. Surfactants have been used extensively in herbicide and pesticide formulations to increase their penetration. However, they must be used judicially, as surfactants may have inhibitory phytotoxic effects on plant tissue at high concentrations and stimulatory effects at low concentrations [1, 2].

One of the most widely known benefits of a surfactant is to boost and simplify the ‘floral dip’ method for *Agrobacterium*-mediated transformation of *Arabidopsis thaliana* [3]. The surfactant, Silwet^®^ L-77, is thought to lower the water surface tension within the intercellular spaces of plant tissue, allowing increased penetration of Agrobacterium through the waxy cuticle and to the floral meristem, where it can effect transformation. The ‘floral dip’ method is popular due to its simplicity compared to the more laborious vacuum infiltration method, allowing large-scale treatment without bulky and expensive equipment or excessive time and effort [3].

Following on the success of Silwet^®^ L-77, newer compounds have become available that may enhance Agrobacterium-mediated transformation of plant tissue. The current study was undertaken to maximize transformation efficiency of the ‘floral dip’ method through a comprehensive evaluation of seven novel surfactants (BREAK-THRU surfactants OE446, S200, S233, S240, S279, S301 and SP133).

## Main text

### Results and discussion

**Fig. 1:**
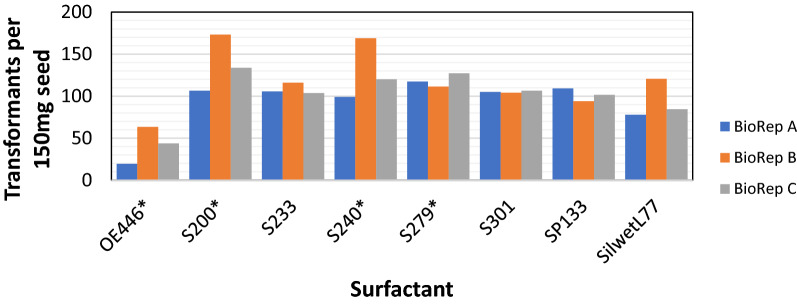
Surfactant transformation efficiency normalized to number of transformants per 150 mg seed. The three sets of Arabidopsis transformations are shown (**A**–**C**)

Three independent batches of Arabidopsis were transformed via floral dip [3] to test transformation efficiency of the seven novel surfactants (BREAK-THRU^®^-OE446, -S200, -S233, -S240, -S279, -S301, and -SP133), which were compared to the commonly used surfactant Silwet^®^ L-77. All surfactant concentrations were held constant at 0.03% (v/v) for the floral dip assays. Approximately 150 mg of seed was plated for each surfactant, with three independent assays per compound. The number of transformants per event was normalized to 150 mg starting weight and rounded to the nearest integer (Fig. 1; Additional file 3: Table S1). Probabilities are compared by Chi-squared tests.

#### Statistical analysis

**Fig. 2:**
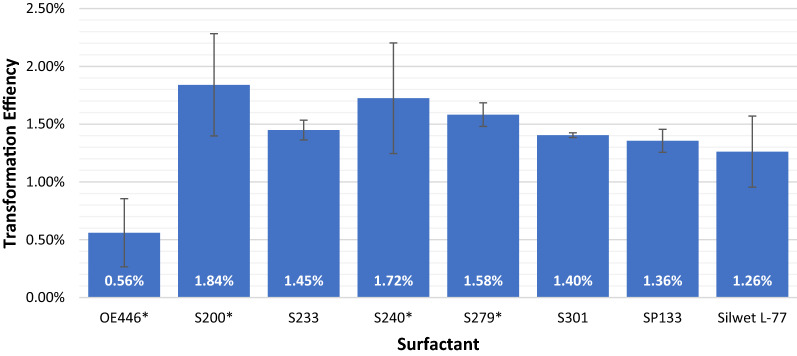
Transformation efficiency normalized to % transformation per 150 mg seed. Values show percent transformation ± standard deviation

Three Arabidopsis floral dipping events were conducted to generate transformed seed to test transformation efficiency of the seven BREAK-THRU^®^ surfactants, compared to the standard surfactant Silwet^®^ L-77. Approximately 150 mg of seeds was plated from each surfactant tested per dipping event and the number of transformants recovered was noted. Results were then normalized to 150 mg starting weight and rounded to the nearest integer. Total number of transformants are reported (see barplot and summary table). Chi-squared tests were performed to compare given probabilities. Significant differences were seen between surfactants (Chi-squared = 170.85, df = 7, p-value < 2.2e−16). Compared to Silwet^®^ L-77, positive significant differences are seen for S200, S240, and S279, whereas OE446 negatively affects transformation efficiency (Additional file 1 Statistics 1: Figure S1, Additional file 3 Statistics 2: Table S2). The remaining surfactants (S233, S301 and SP133) were statistically indistinguishable from the control. Transformation efficiency of independent assays ranged from a high of 2.31% for S200 to a low of 0.25% for OE446 (Additional file 3 Statistics 2: Table S1). Efficiencies for the average of all three data sets were: S200 (1.84%), S240 (1.72%), S279 (1.58%), S233 (1.45%), S301 (1.41%), SSP133 (1.35%), Silwett L77 (1.26%), OE446 (0.56%) (Fig. 2, Additional file 3 Statistics 2: Table S1).

Although surfactants are necessary for efficient transformation, previous reports suggest they can be toxic to plant health leading to necrosis of tissue and reduction of seed yield [4, 5]. In the current study, the tested surfactants produced no observable adverse effects at 0.03% concentration. All seedlings appeared healthy; in fact, S200, S233, and S240 appeared to produce larger and more robust plants than the remaining surfactants, including the control transformation with Silwet^®^ L77.

## Conclusion

Previous research has shown that surfactants such as Tween-20 and Triton-X can be used for Arabidopsis transformation, but the improvement in efficiency is modest at best, and these compounds may have toxicity on the resulting seedlings [4, 5]. The current study has demonstrated that the novel BREAK THRU^®^ surfactants can significantly increase Arabidopsis floral dip transformation efficiency over the commonly used Silwet^®^ L-77 using a standard floral dipping protocol [3]. Three of the surfactants investigated (S200, S240, S279) yielded statically higher transformation efficiencies, three others provided similar efficiencies (S233, S301, SP133), and one (OE446) showed a distinct drop in efficiency when compared to Silwet^®^ L-77 (Figs. 1 and 2, Additional file 3: Table S2). Of all the tested surfactants, S200 consistently performed best, followed closely by S240. Both surfactants generated healthy and robust plants, indicating low levels of phytotoxicity. OE446 was the only compound to show a negative impact on transformation efficiency.

The availability of more surfactants enables further optimization for specific plant transformation. Considerable refinements of current transformation systems are needed to achieve commercial application of transgenics in various crop species. Results presented here may be applied towards these improvements. Surfactants have been shown to improve transformation efficiency for numerous crop species, including but not limited to sugarcane [6], radish [7], soy [8], maize [9], and rice [10, 11]. As these investigations all utilized Silwett^®^ L-77 to enhance transformation, our current results suggest that a 45% increase in the number of transformants may be obtained through the use of BREAK THRU^®^ surfactants S200, S240, and S279.

### Methods

*Arabidopsis thaliana* Columbia-0 (Col-0) seeds were sown in individual pots (four plants per 28 cm^2^ plastic pot) on moist soil (Sunshine Mix#1). Seeds were stratified for 72 h by keeping the sown pots at 4 °C for 3 days. Seventy-two pots of Arabidopsis (Col-0) were prepared, using three pots per floral dip per surfactant. Pots were then transferred to a greenhouse at 22 °C with a photoperiod regime of 16/8 h (long days). When the plants reached ~ 10 cm in height, and the primary inflorescences began to flower, the plants were collected for floral dip transformation [3].

#### Binary vector and *Agrobacterium* culture

Agrobacterium strain AGL1 was transformed with construct pCTAGV-KCN3 [12]. The vector contains the *npt*II gene for kanamycin selection and DSRed for visualization. Three batches of cells were prepared simultaneously, one for each set of transformations. Cells for each batch were grown in 2500 ml of YEP with kanamycin (100 mg/l) at 28 °C in a shaking water bath at 225–250 rpm to OD600 ~ 0.6. Cells were resuspended in infiltration media to OD600 = 0.8 and split into 8 aliquots of 300 ml for addition of each surfactant (below).

#### Arabidopsis transformation

Arabidopsis transformation was conducted using a modified version of the published method [3]. Infiltration media was prepared in 1-l batches, containing 2.2 g MS salts, 1 ml Gamborg 1000× B5 vitamins, 50 g sucrose, and 10 μl 4.4 mg/ml benzylamino purine (BAP) in water. Each surfactant was added to a 300-ml aliquot of prepared cells (above) to a final concentration of 0.03% (v/v). Pots containing the Arabidopsis plants were inverted, and the plants were dipped for 2 min in the infiltration media. After dipping, the pots were laid on their sides, covered with Saran^®^ wrap, and allowed to rest overnight at room temperature. On the following day, the Saran^®^ wrap was removed, and pots were moved back to the greenhouse at 22 °C with a 16/8-h light cycle. Plants were cultivated for 2 weeks with watering, then allowed to dry for 2–3 weeks before seed collection.

#### Selection of putative transformants

Selection of transformants was performed by measuring ~ 50 mg aliquots of seeds from each surfactant-tested batch of plants. Three Agrobacterium cultures were prepared for each of the eight surfactants assayed, giving 24 seed aliquots total. Seeds were surface sterilized by exposure to chlorine gas for 2 h. Once degassed, seeds were placed on selection plates containing MS salts at half strength, supplemented with 50 mg/l kanamycin and solidified by 8 g/l bacto agar. Disinfected seeds were stratified for 72 h at 4 °C, then transferred to a 22 °C growth chamber with a 16/8-h photoperiod. After 7 days, green seedlings with green secondary true leaves were identified as putative transformants to be counted. Ten randomly selected transformed plants from each of the eight surfactants were transplanted to soil mixture (Sunshine Mix #1) for verification of T-DNA transfer by PCR (below; and Additional file 2: Figure S2). Transformation efficiencies were calculated as (number of kanamycin resistant seedlings)/(total number of seeds tested) × 100.

#### Genomic DNA isolation

As a PCR control, Arabidopsis genomic DNA was isolated using the ‘Gentra PureGene DNA isolation kit’ protocol (Qiagen), as described by the manufacturer. Endpoint PCR amplification was performed with the primers described below.

#### Polymerase chain reaction

PCR was performed to verify integration of the T-DNA region into the plant genome. Small leaf segments were collected from plants 2 weeks after transplantation, and genomic DNA was isolated (above). One microliter of gDNA (10–50 ng) was used as template. A 628-bp fragment of the *npt*II gene in the T-DNA region was amplified with Forward primer NPTII F57 (5′-GATTGAACAAGATGGATTGCACGC) and Reverse primer NPTII R58 (5′-CCACAGTCGATGAATCCAGAAAAGC) for 32 cycles and an annealing temperature of 58 °C. See Additional File 2 for representative PCR results.

#### Control surfactant

Silwet^®^ L-77: Trisiloxane nonionic organosilicone surfactant co-polymer, with enhanced wetting and spreading characteristics when used in aqueous sprays [13].

#### Tested surfactants [14]

BREAK THRU^®^-OE446: Polysiloxane (oil and water solubility), stable within a pH range of 3–11, spreader specially formulated for methylated vegetable oils, anti-dust agent in WDG and for seed treatments; Static surface tension [mN/m] in water: 25 (0.1%).

BREAK THRU^®^-S200: Trisiloxane super-spreader (more water-soluble), stable within pH range 6–8, Static surface tension [mN/m] in water: 22 (0.1%).

BREAK THRU^®^-S233: Trisiloxane super-penetrant (enhances biological performance of (semi-) systemic products, stable within pH range 6–8, Static surface tension [mN/m] in water: 23 (0.1%).

BREAK THRU^®^-S240: Trisiloxane super-spreader (liquid soluble), stable within pH range 6–8, Static surface tension [mN/m] in water: 22 (0.1%).

BREAK THRU^®^-S279: Trisiloxane super-spreader (more oil-soluble), stable within pH range 6–8, Static surface tension [mN/m] in water: 21 (0.1%).

S301: Trisiloxane super-spreader, stable within pH range 6–8, Static surface tension [mN/m] in water: 22 (0.1%).

BREAK THRU^®^-SP133: Polyglycerol ester-based adjuvant (reduces the number of particles prone to drift, low foam tendency), stable within pH range 4–9, Static surface tension [mN/m] in water: 29 (0.1%).

For more details of the BREAK THRU^®^ compound see (www.evonik.com/break-thru) [14].

## Limitations

We show an increase in transformation efficiency for Arabidopsis floral dip transformation using a published technique [3]. However, it is speculated, but not proven that tissue culture transformation efficiencies could be improved by optimizing the type and amount of surfactant utilized and length of time used.

## Supplementary Information

**Additional file 1. Statistics 1: Figure S1.**

**Additional file 2. PCR: Figure S2.**

**Additional file 3. Statistics 2: Table S1. **Surfactant transformation efficiency. **Table S2.** Surfactant transformation statistics.

## Data Availability

All data and material are available upon request; please contact corresponding author James.Thomson@USDA.GOV. Mention of trade names or commercial products is solely for the purpose of providing specific information and does not imply recommendation or endorsement by the US Department of Agriculture. USDA is an equal opportunity provider and employer.

## Abbreviation

T-DNA: Transfer DNA originating from an Agrobacterium and shuttled into a plant cell for integration into the host genome

## References

[1] Rinallo C, Bennici A, Cenni E (1988). Effects of two surfactants on Triticum durum Deft. plantlets. Environ Exp Bot.

[2] Bruschi P, Schiff S, Bennici A, Mori B (1998). An example of in vitro test to study the effects of surfactants in plant materials. Chemosphere.

[3] Clough SJ, Bent AF (1998). Floral dip: a simplified method for Agrobacterium-mediated transformation of *Arabidopsis*
*thaliana*. Plant J Cell Mol Biol.

[4] Dehestani A, Ahmadian G, Salmanian AH, Jelodar NB, Kazemitabar K (2010). Transformation efficiency enhancement of Arabidopsis vacuum infiltration by surfactant application and apical inflorescence removal. Trakia J Sci.

[5] Kim MJ, Baek K, Park C-M (2008). Optimization of conditions for transient Agrobacterium-mediated gene expression assays in Arabidopsis. Plant Cell Rep.

[6] Mayavan S, Subramanyam K, Arun M, Rajesh M, Kapil Dev G, Sivanandhan G, Jaganath B, Manickavasagam M, Selvaraj N, Ganapathi A (2013). Agrobacterium tumefaciens-mediated in planta seed transformation strategy in sugarcane. Plant Cell Rep.

[7] Curtis IS, Nam HG (2001). Transgenic radish (*Raphanus sativus* L. longipinnatus Bailey) by floral-dip method—plant development and surfactant are important in optimizing transformation efficiency. Transg Res.

[8] Li S-J, Wei Z-M, Huang J-Q (2008). The effect of co-cultivation and selection parameters on Agrobacterium-mediated transformation of Chinese soybean varieties. Plant Cell Rep.

[9] Chen L, Cong Y, He H, Yu Y (2014). Maize (*Zea mays* L.) transformation by *Agrobacterium tumefaciens* infection of pollinated ovules. J Biotechnol.

[10] Andrieu A, Breitler JC, Siré C, Meynard D, Gantet P, Guiderdoni E (2012). An in planta, Agrobacterium-mediated transient gene expression method for inducing gene silencing in rice (*Oryza sativa* L.) leaves. Rice.

[11] Burman N, Chandran D, Khurana JP (2020). A rapid and highly efficient method for transient gene expression in rice plants. Plants Front Plant Sci.

[12] de Oliveira ML, Stover E, Thomson JG (2015). The *codA* gene as a negative selection marker in *Citrus*. Springerplus.

[13] Silwet L-77. n.d. https://helenaagri.com/products/utility/silwet-l77/. Accessed 05 Mar 2021.

[14] BREAK-THRU^®^—SURFACTANT technology FROM EVONIK. n.d. https://www.break-thru.com/product/break-thru/en. Accessed 24 Feb 2021.

