# Supplementary material for: Evaluation of novel surfactants for plant transformation
Source: BMC Res Notes. 2022 Dec 8;15:360. doi: 10.1186/s13104-022-06251-5 (PMC9733262; doi:10.1186/s13104-022-06251-5)
Supplement: Supplementary file 1 — Additional file 1. Statistics 1: Figure S1. [file 13104_2022_6251_MOESM1_ESM.pdf]

20210220 Surf Round150

```
#In this expt, 3 batches of AGRO + 8 surfactants. Expt done in technical triplicate
(separate weight and sowing) + biological triplicate. Decision made to combine techni
cal replicates and normalize to 150mg seed, instead of 3 x 50mg. Final count number i
s rounded to nearest integer.

#Doing comparison of all surfactants to SilwetL77

#Here dealing with normalized count data, for 150mg seed, rounded to nearest integer.


#Looking at Surf

library(readr)
Surf <- read_csv("~/Desktop/Protocols/Thomson lab/Papers_2021/20210220_Surfactants/Su
rfactants_R20210220.csv")
```

```
## Parsed with column specification:
## cols(
##   Surfactant = col_character(),
##   BioRep = col_character(),
##   Count150 = col_double(),
##   Round150 = col_double()
## )
```

```
#View(Surf)

Surf$Surfactant = factor(Surf$Surfactant, levels=unique(Surf$Surfactant))
attach(Surf)
names(Surf)
```

```
## [1] "Surfactant" "BioRep"     "Count150"   "Round150"
```

```
str(Surf)
```

```
## tibble [24 × 4] (S3: spec_tbl_df/tbl_df/tbl/data.frame)
##  $ Surfactant: Factor w/ 8 levels "OE446","S200",..: 1 2 3 4 5 6 7 8 1 2 ...
##  $ BioRep    : chr [1:24] "A" "A" "A" "A" ...
##  $ Count150  : num [1:24] 19.4 106.6 105.6 99 117.4 ...
##  $ Round150  : num [1:24] 19 107 106 99 117 105 109 78 63 173 ...
##  - attr(*, "spec")=
##   .. cols(
##   ..   Surfactant = col_character(),
##   ..   BioRep = col_character(),
##   ..   Count150 = col_double(),
##   ..   Round150 = col_double()
##   .. )
```

```
library(ggplot2)
library(dplyr)
```

```
##
## Attaching package: 'dplyr'
```

```
## The following objects are masked from 'package:stats':
##
##     filter, lag
```

```
## The following objects are masked from 'package:base':
##
##     intersect, setdiff, setequal, union
```

```
#Summary counts by biorep
ggplot(Surf, aes( x = Surfactant, y = Round150, shape=BioRep)) + geom_point(size=5) +
theme_bw() + theme(axis.title= element_text(face="bold")) + ylab("Transformants per 1
50mg seed")
```

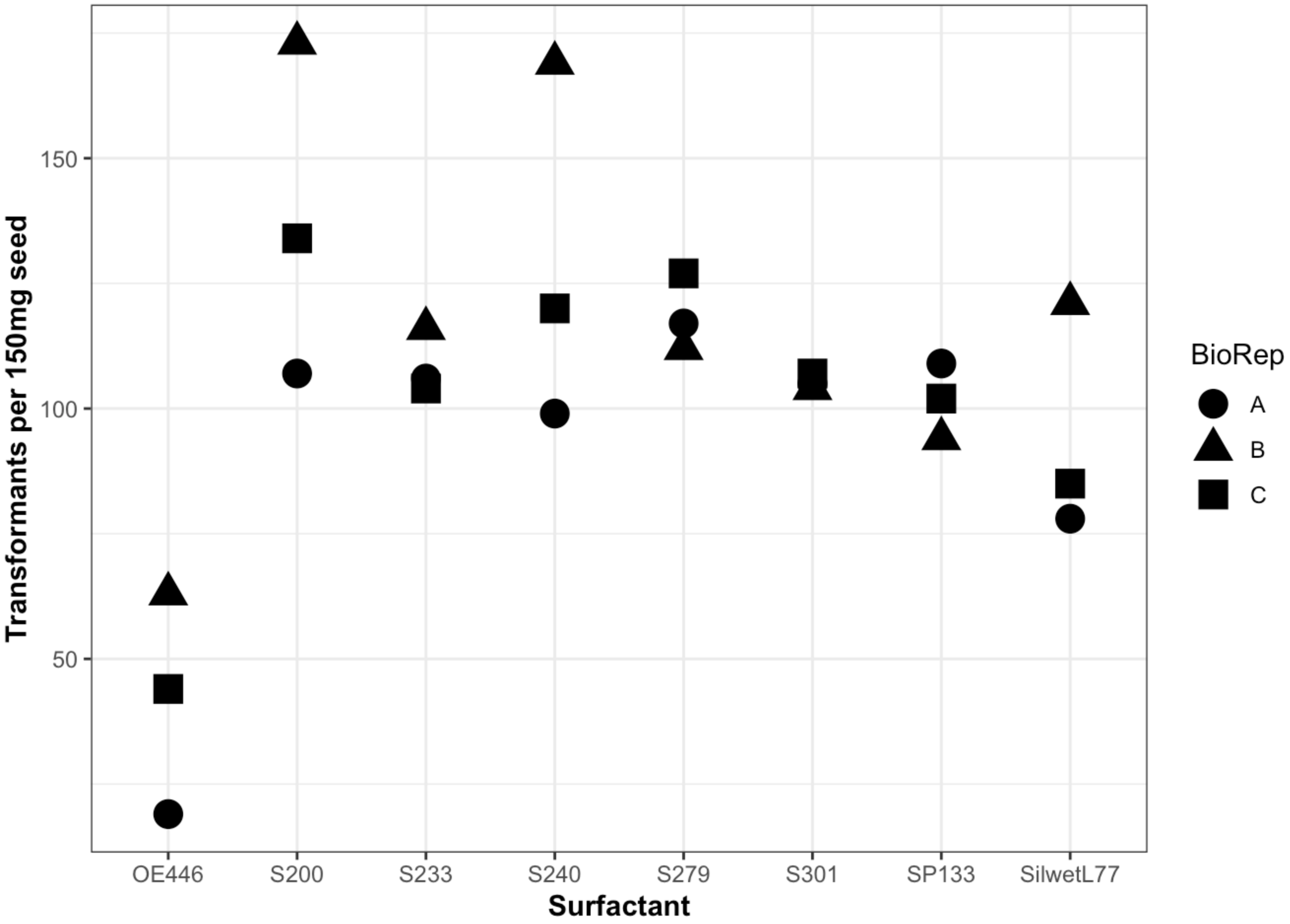


```
ggsave("20210221_PlotInd_RND.png")
```

```
## Saving 7 x 5 in image
```

```
#Summary counts by surfactant

Surf2 <- Surf %>% group_by(Surfactant) %>% summarize(SumCount = sum(Round150))
```

```
## `summarise()` ungrouping output (override with `.groups` argument)
```

```
#View(Surf2)

ggplot(Surf2) + aes(x=Surfactant, y=SumCount) + geom_bar(stat="identity") + geom_text
(aes(label = SumCount, y = SumCount), vjust = -0.1) + theme_bw() + theme(axis.title=
element_text(face="bold")) + ylab("Number of Transformants")
```

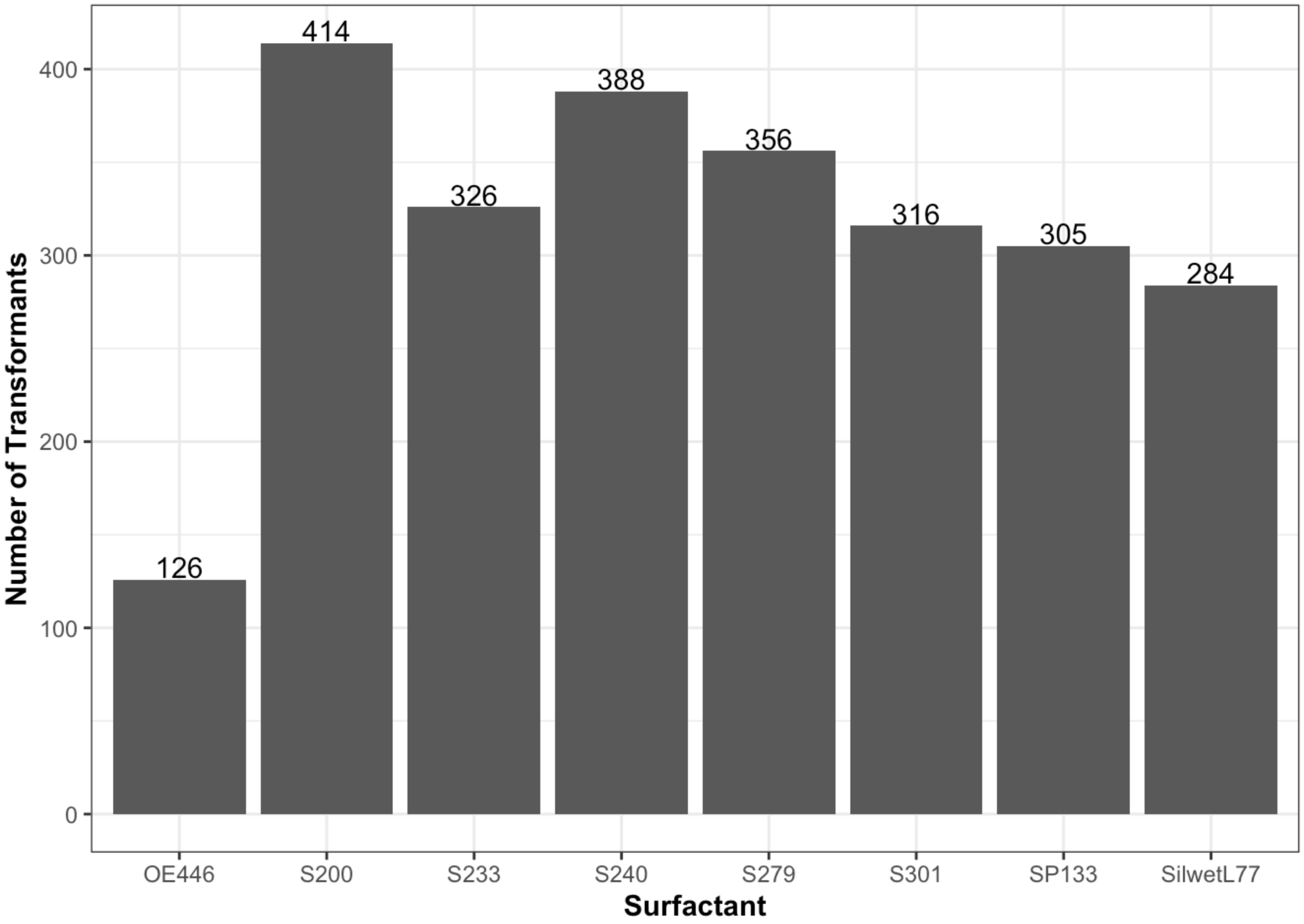


```
ggsave("20210307_Plot_SumCount.png")
```

```
## Saving 7 x 5 in image
```

```
#Chi-sq
Tabla = xtabs(Round150~Surfactant)
Tabla
```

```
## Surfactant
##     OE446      S200      S233      S240      S279      S301     SP133 SilwetL77
##       126       414       326       388       356       316       305       284
```

```
chisq.test(Tabla)
```

```
##
##  Chi-squared test for given probabilities
##
## data:  Tabla
## X-squared = 170.85, df = 7, p-value < 2.2e-16
```

```
OE446= sum(Surf$Round150[Surf$Surfactant=="OE446"])
OE446
```

```
## [1] 126
```

```
S200= sum(Surf$Round150[Surf$Surfactant=="S200"])
S200
```

```
## [1] 414
```

```
S233= sum(Surf$Round150[Surf$Surfactant=="S233"])
S233
```

```
## [1] 326
```

```
S240= sum(Surf$Round150[Surf$Surfactant=="S240"])
S240
```

```
## [1] 388
```

```
S279= sum(Surf$Round150[Surf$Surfactant=="S279"])
S279
```

```
## [1] 356
```

```
S301= sum(Surf$Round150[Surf$Surfactant=="S301"])
S301
```

```
## [1] 316
```

```
SP133= sum(Surf$Round150[Surf$Surfactant=="SP133"])
SP133
```

```
## [1] 305
```

```
SilwetL77= sum(Surf$Round150[Surf$Surfactant=="SilwetL77"])
SilwetL77
```

```
## [1] 284
```

```
#OE446 to SilwetL77
observed = c(OE446, SilwetL77)   # observed frequencies
expected = c(1/2, 1/2)             # expected proportions

chi1 = chisq.test(x = observed,
           p = expected)

chi1
```

```
##
##  Chi-squared test for given probabilities
##
## data:  observed
## X-squared = 60.888, df = 1, p-value = 6.042e-15
```

```
chi1$p.value
```

```
## [1] 6.042198e-15
```

```
chi1$statistic
```

```
## X-squared
##   60.8878
```

```
chi1$parameter
```

```
## df
##  1
```

```
#S200 to SilwetL77
observed = c(S200, SilwetL77)   # observed frequencies
expected = c(1/2, 1/2)             # expected proportions

chi2 = chisq.test(x = observed,
           p = expected)

chi2
```

```
##
##  Chi-squared test for given probabilities
##
## data:  observed
## X-squared = 24.212, df = 1, p-value = 8.629e-07
```

```
chi2$p.value
```

```
## [1] 8.62914e-07
```

```
#S233 to SilwetL77
observed = c(S233, SilwetL77)   # observed frequencies
expected = c(1/2, 1/2)             # expected proportions

chi3 = chisq.test(x = observed,
           p = expected)

chi3
```

```
##
##  Chi-squared test for given probabilities
##
## data:  observed
## X-squared = 2.8918, df = 1, p-value = 0.08903
```

```
chi3$p.value
```

```
## [1] 0.08903122
```

```
#S240 to SilwetL77
observed = c(S240, SilwetL77)   # observed frequencies
expected = c(1/2, 1/2)             # expected proportions

chi4 = chisq.test(x = observed,
           p = expected)

chi4
```

```
##
##  Chi-squared test for given probabilities
##
## data:  observed
## X-squared = 16.095, df = 1, p-value = 6.024e-05
```

```
chi4$p.value
```

```
## [1] 6.023531e-05
```

```
#S279 to SilwetL77
observed = c(S279, SilwetL77)   # observed frequencies
expected = c(1/2, 1/2)             # expected proportions

chi5 = chisq.test(x = observed,
           p = expected)

chi5
```

```
##
##  Chi-squared test for given probabilities
##
## data:  observed
## X-squared = 8.1, df = 1, p-value = 0.004427
```

```
chi5$p.value
```

```
## [1] 0.004426526
```

```
#S301 to SilwetL77
observed = c(S301, SilwetL77)   # observed frequencies
expected = c(1/2, 1/2)             # expected proportions

chi6 = chisq.test(x = observed,
           p = expected)

chi6
```

```
##
##  Chi-squared test for given probabilities
##
## data:  observed
## X-squared = 1.7067, df = 1, p-value = 0.1914
```

```
chi6$p.value
```

```
## [1] 0.1914184
```

```
#SP133 to SilwetL77
observed = c(SP133, SilwetL77)   # observed frequencies
expected = c(1/2, 1/2)             # expected proportions

chi7 = chisq.test(x = observed,
           p = expected)

chi7
```

```
##
##  Chi-squared test for given probabilities
##
## data:  observed
## X-squared = 0.74873, df = 1, p-value = 0.3869
```

```
chi7$p.value
```

```
## [1] 0.3868797
```

```
Pvalue <- c(chi1$p.value,chi2$p.value,chi3$p.value,chi4$p.value,chi5$p.value,chi6$p.v
alue,chi7$p.value)
Pvalue
```

```
## [1] 6.042198e-15 8.629140e-07 8.903122e-02 6.023531e-05 4.426526e-03
## [6] 1.914184e-01 3.868797e-01
```

```
Padj <- p.adjust(Pvalue, method = "holm")
Padj
```

```
## [1] 4.229538e-14 5.177484e-06 2.670937e-01 3.011765e-04 1.770610e-02
## [6] 3.828369e-01 3.868797e-01
```

```
df <- c(chi1$parameter,chi2$parameter,chi3$parameter,chi4$parameter,chi5$parameter,ch
i6$parameter,chi7$parameter)
df
```

```
## df df df df df df df
##  1  1  1  1  1  1  1
```

```
Xsquared <- c(chi1$statistic,chi2$statistic,chi3$statistic,chi4$statistic,chi5$statis
tic,chi6$statistic,chi7$statistic)
Xsquared
```

```
##  X-squared  X-squared  X-squared  X-squared  X-squared  X-squared  X-squared
## 60.8878049 24.2120344  2.8918033 16.0952381  8.1000000  1.7066667  0.7487267
```

```
SurfN <- as.vector(unique(Surf$Surfactant[1:7]))
SurfN
```

```
## [1] "OE446" "S200"  "S233"  "S240"  "S279"  "S301"  "SP133"
```

```
Summary <- cbind(SurfN, Xsquared, df, Pvalue, Padj)
Summary
```

```
##           SurfN   Xsquared            df  Pvalue
## X-squared "OE446" "60.8878048780488"  "1" "6.04219766673002e-15"
## X-squared "S200"  "24.2120343839542"  "1" "8.62913981113324e-07"
## X-squared "S233"  "2.89180327868852"  "1" "0.0890312232637878"
## X-squared "S240"  "16.0952380952381"  "1" "6.02353074228851e-05"
## X-squared "S279"  "8.1"               "1" "0.00442652585791983"
## X-squared "S301"  "1.70666666666667"  "1" "0.191418425237607"
## X-squared "SP133" "0.748726655348047" "1" "0.38687967889819"
##           Padj
## X-squared "4.22953836671101e-14"
## X-squared "5.17748388667994e-06"
## X-squared "0.267093669791363"
## X-squared "0.000301176537114425"
## X-squared "0.0177061034316793"
## X-squared "0.382836850475214"
## X-squared "0.38687967889819"
```

```
write.csv(Summary, "20210307_SummaryFinal_Surf.csv")
```
