# Supplementary material for: Evaluation of novel surfactants for plant transformation
Source: BMC Res Notes. 2022 Dec 8;15:360. doi: 10.1186/s13104-022-06251-5 (PMC9733262; doi:10.1186/s13104-022-06251-5)

PCR verification of transformation

Silwet L-77

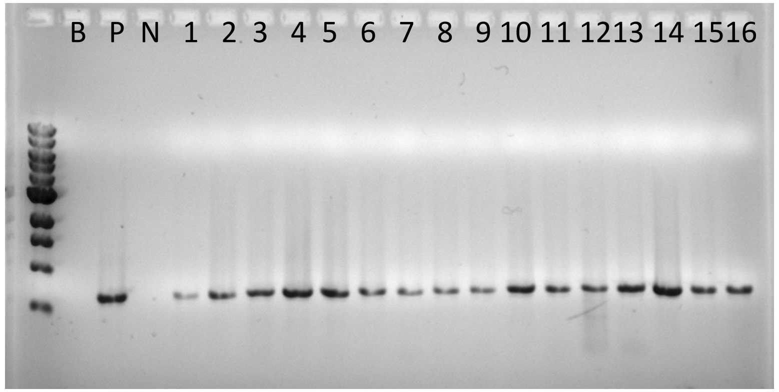

OE446

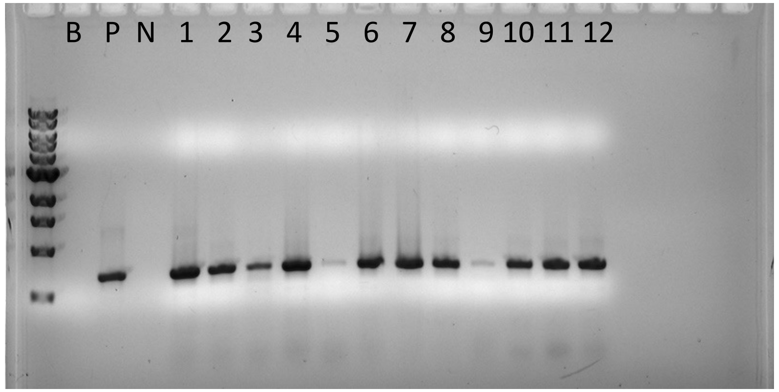

S200

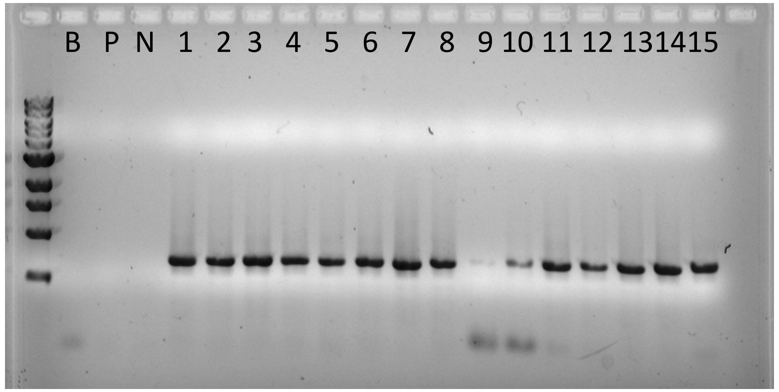

S233

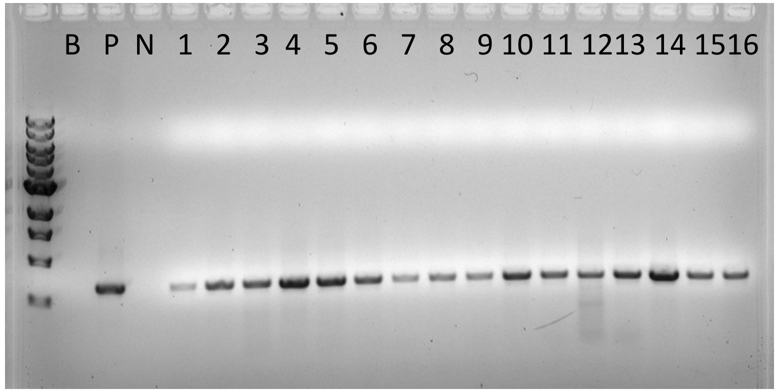

Thermocycler conditions:

94oC 2 min (1 cycle)

94oC 30 sec
57oC 30 sec
72oC 30 sec

Primers:
NPTII F57 5’ GATTGAACAAGATGGATTGCACGC
NPTII R58 5’ CCACAGTCGATGAATCCAGAAAAGC

Amplicon size 628 bp

PCR verification of transformation

S240

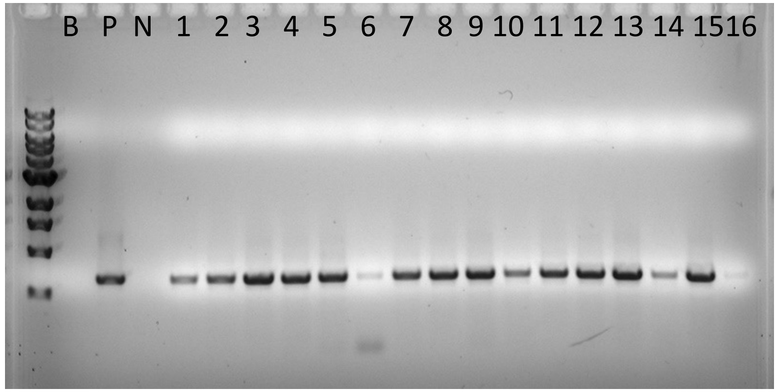

S279

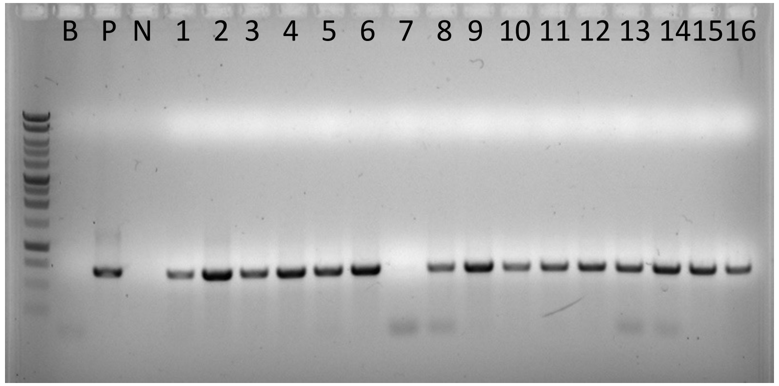

Thermocycler conditions:

94oC 2 min (1 cycle)

94oC 30 sec
57oC 30 sec
72oC 30 sec

Primers:
NPTII F57 5’ GATTGAACAAGATGGATTGCACGC
NPTII R58 5’ CCACAGTCGATGAATCCAGAAAAGC

Amplicon size 628 bp

S301

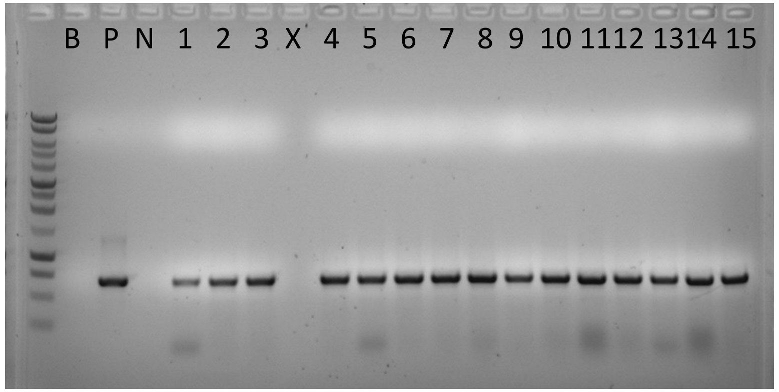

SP133

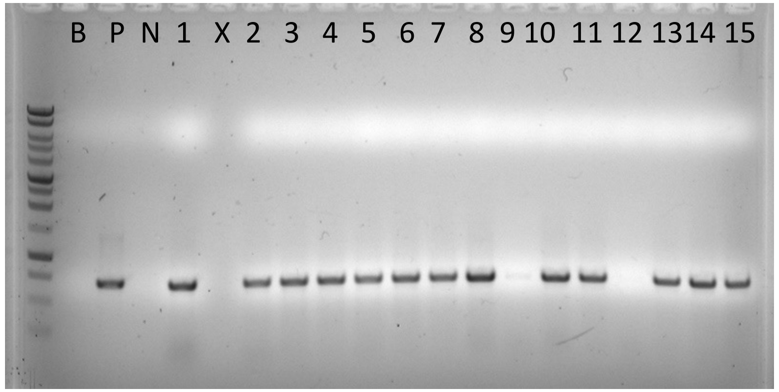

Supplement: Supplementary file 2 — Additional file 2. PCR: Figure S2. [file 13104_2022_6251_MOESM2_ESM.pdf]
